# T-tubule biogenesis and triad formation in skeletal muscle and implication in human diseases

**DOI:** 10.1186/2044-5040-1-26

**Published:** 2011-07-13

**Authors:** Lama Al-Qusairi, Jocelyn Laporte

**Affiliations:** 1 Department of Translational Medecine and Neurogenetics, IGBMC (Institut de Génétique et de Biologie Moléculaire et Cellulaire), 1 rue Laurent Fries, 67404 Illkirch, France; 2 Inserm, U964, Illkirch, 1 rue Laurent Fries, 67404, France; 3 CNRS, UMR7104, 1 rue Laurent Fries, 67404, Illkirch, France; 4 Université de Strasbourg, 1 rue Laurent Fries, 67404, Illkirch, France; 5 Collège de France, chaire de génétique humaine, 1 rue Laurent Fries, 67404 Illkirch, France; 6 Département de Pharmacologie & Toxicologie, Université de Lausanne, 27 rue du Bugnon, 1005 Lausanne, Switzerland

## Abstract

In skeletal muscle, the excitation-contraction (EC) coupling machinery mediates the translation of the action potential transmitted by the nerve into intracellular calcium release and muscle contraction. EC coupling requires a highly specialized membranous structure, the triad, composed of a central T-tubule surrounded by two terminal cisternae from the sarcoplasmic reticulum. While several proteins located on these structures have been identified, mechanisms governing T-tubule biogenesis and triad formation remain largely unknown. Here, we provide a description of triad structure and plasticity and review the role of proteins that have been linked to T-tubule biogenesis and triad formation and/or maintenance specifically in skeletal muscle: caveolin 3, amphiphysin 2, dysferlin, mitsugumins, junctophilins, myotubularin, ryanodine receptor, and dihydhropyridine Receptor. The importance of these proteins in triad biogenesis and subsequently in muscle contraction is sustained by studies on animal models and by the direct implication of most of these proteins in human myopathies.

## Introduction

**Figure 1:**
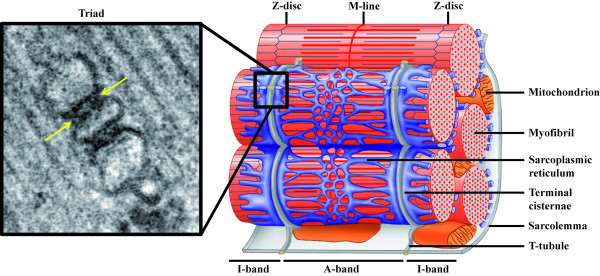
**Triad organization in skeletal muscle**. Left: Electron micrograph of a triad junction. A central T-tubule is flanked on both sides by a terminal cisternae element from the sarcoplasmic reticulum. Arrows indicate electron-dense junctional feet corresponding to the ryanodine receptor-dihydhropyridine receptor complex. Right: Schematic representation of a mammalian muscle sarcomere and surrounding membranes. T-tubules shown in gray are specialized invaginations of the sarcolemma. The elaborated sarcoplasmic reticulum network is shown in blue. Note the close proximity of T-tubules and terminal cisternae of the sarcoplasmic reticulum (adapted from [104]; ^© ^2007 by Pearson Education, Inc.).

To trigger skeletal muscle contraction, the action potential generated by motor neurons is transmitted through motor nerves to muscle cells. The excitation-contraction (EC) coupling, *i.e*. signal transmission from the sarcolemma to the actin/myosin apparatus, is mediated by a second messenger, calcium ions. Indeed, muscle fibers contain large internal calcium stores with the ability to quickly release and retrieve calcium (Figure 1, right panel). For a fast and fine-tuning of muscle contraction, these stores are maintained under the control of the action potential, which ensures calcium release simultaneously within the whole interior of the muscle fiber. As myofibers are 50-100 μm in diameter and several millimeters to centimeters long, a highly specialized structure named the triad is necessary to overcome spatial limits in using calcium as secondary messenger, and connect the sarcolemma with the calcium stores. The sarcolemma forms regular invaginations which insert between myofibrils, termed transverse tubules (T-tubules). In skeletal muscle, T-tubules tightly associate with the sarcoplasmic reticulum (SR), in a region called terminal cisternae/junctional SR. The close association of one T-tubule with two terminal cisternae on both sides of the tubule forms the triad (Figure 1).

A large set of specialized proteins takes part in EC coupling and includes: i) the Dihydhropyridine Receptor (DHPR), a voltage gated calcium channel located on T-tubule membranes [1,2], ii) the Ryanodine Receptor (RyR1), a calcium release channel that is localized on the junctional face of SR and appears as "feet" when observed by electron microscopy (Figure 1, left panel) [3], iii) calcium buffering proteins such as calsequestrin in the lumen of the SR [4], iv) calcium channel regulators such as calmodulin, FKBP12 and many others [5,6], v) Sarco-Endoplasmic Reticulum Calcium ATPase pumps (SERCA), which is indirectly involved in EC coupling via its action in the rapid removal of the cytosolic calcium after fiber shortening to replenish the calcium stores [7]. Noteworthy, the physical coupling between RyR1 and DHPR occurs specifically in skeletal but not in cardiac muscles and allows the transmission of the signal within 2 ms in skeletal muscles compared to 100 ms in cardiac muscles [8]. In cardiac myofiber, RyR2-mediated calcium release is induced by extracellular calcium entry via DHPR in a mechanism called calcium-induced calcium release (CICR) [8]. Moreover, in fibers of small diameter, such as the body muscles of Amphioxus, peripheral couplings between SR and the plasmalemma have similar function to triads. In addition, all differentiating muscle fibers pass through a stage where T-tubules are not present and EC coupling is mediated by such peripheral couplings.

In this review, we will focus on the molecular mechanisms underlying T-tubules biogenesis and triad formation specifically in skeletal muscle. Triad defects linked to human monogenic diseases will also be highlighted.

### T-tubule plasticity

The T-tubule membrane possesses a high plasticity which provides the stability required during muscle contraction, and facilitates repair upon damage. In addition to its principal function in EC coupling, the plasticity of T-tubules confers to this system non-related EC functions.

**Figure 2:**
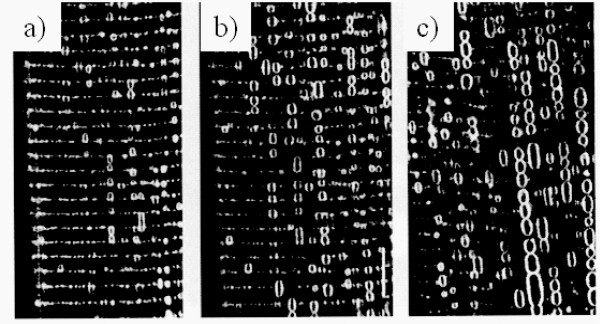
**Dynamics of T-tubule vacuolation produced by the efflux of glycerol**. A single frog skeletal muscle fiber stained with a lipophilic fluorescence probe is shown. Shown are serial confocal microscopic images of the same fiber **(a) **5 minutes, **(b) **12 minutes and **(c) **30 minutes after the fiber was transferred from a solution containing 110 mM glycerol to a solution without glycerol (reprinted from [10]; ^© ^2001 with permission from Elsevier).

It has been reported that the treatment of isolated muscle fibers with glycerol efflux-influx or with other low molecular weight nonelectrolytes (such as sugars) physically affects T-tubules morphology. Such osmotic shock can convert the T-tubule network into many membrane-bound vacuoles, which can either remain interconnected by normal T-tubules, or become separated (Figure 2) [9,10]. Surprisingly, this vacuolation which results in 5-15 fold increase in the relative volume of T-tubule system is reversed spontaneously [11,12]. Moreover, this observation is specific to transverse tubule membranes, as no other intracellular membrane systems appear to be involved [12], probably due to the fact that their lumen connects to the extracellular space.

In addition to this artificial condition, this vacuolation phenomenon is observed upon muscle fatigue or diseases [10,13]. Based on this plasticity and on the large membrane surface of the T-tubules network which corresponds to about 80% of the sarcolemma surface, several functions non-related to EC coupling are proposed for the T-tubules system [10,13]. These include: i) water balance and regulation of cell volume, ii) recovery from muscle fatigue, iii) transport pathways including endocytosis, exocytosis and the penetration of foreign DNA. The molecular mechanisms involved in these processes are still to be investigated.

### Morphological aspects of triad biogenesis

#### Sarcoplasmic reticulum

The SR represents the main calcium store in striated muscle. It is highly specialized to ensure the simultaneous release of intracellular calcium in the entire cytosol of the muscle cell. The first step of SR biogenesis starts by the formation of tubular endoplasmic reticulum (ER) (30-60 nm in diameter) adjacent to the myofibril [14]. Subsequently, these tubular branches of ER develop into reticular structures surrounding the myofibrils [15]. Finally, the newly formed SR engages couplings at the A-I interface with the T-tubule originating from the sarcolemma. The molecular determinants implicated in the functional and structural organization of the SR have been reviewed elsewhere [16].

The chronology of SR biogenesis was well investigated using electron microscopy (EM) during muscle differentiation in mouse [17]. These observations were also supported by studies employing chicken embryo [18]. In mouse, the SR is detected from as early as embryonic day 14 (E14) with punctate RyR clusters that are located in the periphery of the myofiber [17]. At this stage, the content of the feet (RyR) in the junctional SR is poor, and some SR elements without any feet are observed. At E16, RyR containing elements become abundant and start to be associated with the edges of A bands (A-I junctions) of the newly formed sarcomeres. This association results in a distinct banding pattern of a discrete SR network at the I-band with thin longitudinal connecting SR elements [17]. During the next days (E17 and E18), junctional SR acquires a predominant transverse distribution taking their final position by forming triad rows at each side of the Z-line (two SR sacs connecting one T-tubule in each triad) [17]. During the maturation of SR membranes, the frequency of feet increases, in particular, between E16 and E18, when all junctions become filled by feet. The width of the junctional gap is between 9 and 12 nm. The maturation of the RyR containing elements is accomplished at birth [17]. Elegant experiments using tagged SR proteins in differentiating myotubes showed that the SR organization was paralleled by a dynamic localization of longitudinal and junctional SR proteins [19].

#### Transverse tubule

T-tubules are invaginations of the plasma membrane, which are present exclusively in striated muscle. Their role is to maintain the SR calcium store under the tight control of membrane depolarization via the voltage sensor channel DHPR [2]. Morphological studies in chicken and mouse embryos have revealed that the T-tubules start their formation after the SR [17,18]. In mouse embryos, the first defined tubules can be observed at E15. At this stage they take the aspect of short cylinders invaginating from the sarcolemma within the myotubes [17]. At E16, the newly formed T-tubules extend deeper within the myofiber, maintaining a connection with the surface by short transverse segments however they stay predominantly longitudinal. During the last days of gestation (E17, 18, 19), T-tubules progressively invade the entire fiber; the majority of them are longitudinal with some transverse connecting elements [17,18]. The transverse orientation of T-tubules is achieved during the postnatal period. Final maturation of T-tubules is completed in mouse 3 weeks after birth [17,20].

### Molecular mechanisms involved in T-tubule biogenesis and triad formation and maintenance

Although the events characterizing T-tubule biogenesis and triad formation are morphologically defined, molecular mechanisms remain elusive. In the last 20 years, several proteins were proposed to be involved in these mechanisms, and mutations within most of the corresponding genes are associated to muscular disorders in human and/or in rodents. These proteins include caveolin 3 (CAV3), the skeletal muscle specific isoform of amphiphysin 2 (BIN1), dysferlin (DYSF), mitsugumins (MG), junctophilin (JPH1), and myotubularin (MTM1) (Table 1). In this context, one should distinguish between mechanisms leading to T-tubule biogenesis and those involved in the proper assembling of triad components (i.e. one T-tubule element with two junctional SR cisternae). Based on previous reports, we classified CAV3, BIN1, DYSF in T-tubule biogenesis, and mitsugumins and junctophilin in triad formation, while the position of MTM1 in this classification is still unclear.

**Table 1:** Proteins implicated in triad organization, roles and associated human diseases^a^

	Phenotype in humans		Phenotype in rodents				
					
Proteins	Skeletal muscle	Cardiac muscle	Skeletal muscle	Cardiac muscle	Membrane binding	Membrane events	Associated human diseases
CAV3	Yes	Yes	Yes	Yes	Cholesterol binding	Caveolae formation	LGMD-1C, RMD, FHC, LQTS
BIN1	Yes	Yes	n.r.	Yes	Phosphoinositide binding	Membrane tubulation	ARCNM
DYSF	Yes	Yes	Yes	Yes	Transmembrane/phospholipid binding	Membrane repair	Dysferlinopathies
MG29	n.r.	n.r.	Yes	No	Transmembrane binding	Triad structure	n.r.
JPH1	n.r.	n.r.	Yes	No	Phospholipid binding	Sarcolemma and SR junction formation	n.r.
MTM1	Yes	No	Yes	No	Phosphoinositide binding	Membrane transport	XLMTM (XLCNM)

^a^n.r., not reported; LGMD-1C, limb-girdle muscular dystrophy type 1C; RMD, rippling muscle disease; FHC, familial hypertrophic cardiomyopathy; LQTS, long-QT syndrome; ARCNM, autosomal recessive centronuclear myopathy; SR, sarcoplasmic reticulum; XLMTM, X-linked myotubular myopathy; XLCNM, X-linked centronuclear myopathy.

#### Caveolin 3

Caveolae are subcompartments of the plasma membrane which take the aspect of 50-100 nm vesicular invaginations, and have an important role in signal transduction and vesicular transport [21]. In contrast to the other plasma membrane regions which are composed mainly of phospholipids, caveolae are considered as cholesterol-sphingolipid rich raft domains [22]. The principal protein components of the caveolae are the caveolins (CAV), which are cholesterol-binding proteins [22]. The caveolin family is represented in mammals by three members; both CAV1 and CAV2 are co-expressed in non-muscle cells especially adipocytes [21,23] whereas CAV3 is found essentially in striated muscles, and its expression is induced during muscle differentiation [24]. In skeletal muscle, CAV3 localizes at the sarcolemma where it can form a complex with dystrophin and its associated glycoproteins [25]. In addition to the sarcolemma, CAV3 is localized to the developing T-tubules [26].

Mutations within *CAV3 *are associated with several muscular disorders. In particular, mutations which lead to loss of the CAV3 protein, or a decrease of more than 90% of CAV3 expression, result in autosomal dominant limb-girdle muscular dystrophy (LGMD1C), manifesting by mild to moderate proximal muscle weakness [27]. In addition, *CAV3 *is found mutated in rippling muscle disease [28], familial hypertrophic cardiomyopathy [29] and long QT syndrome 9 [30]. Moreover, its expression is increased in tibialis anterior from the mdx mouse, suggesting that CAV3 may contribute to the pathogenesis of DMD [31].

**Figure 3:**
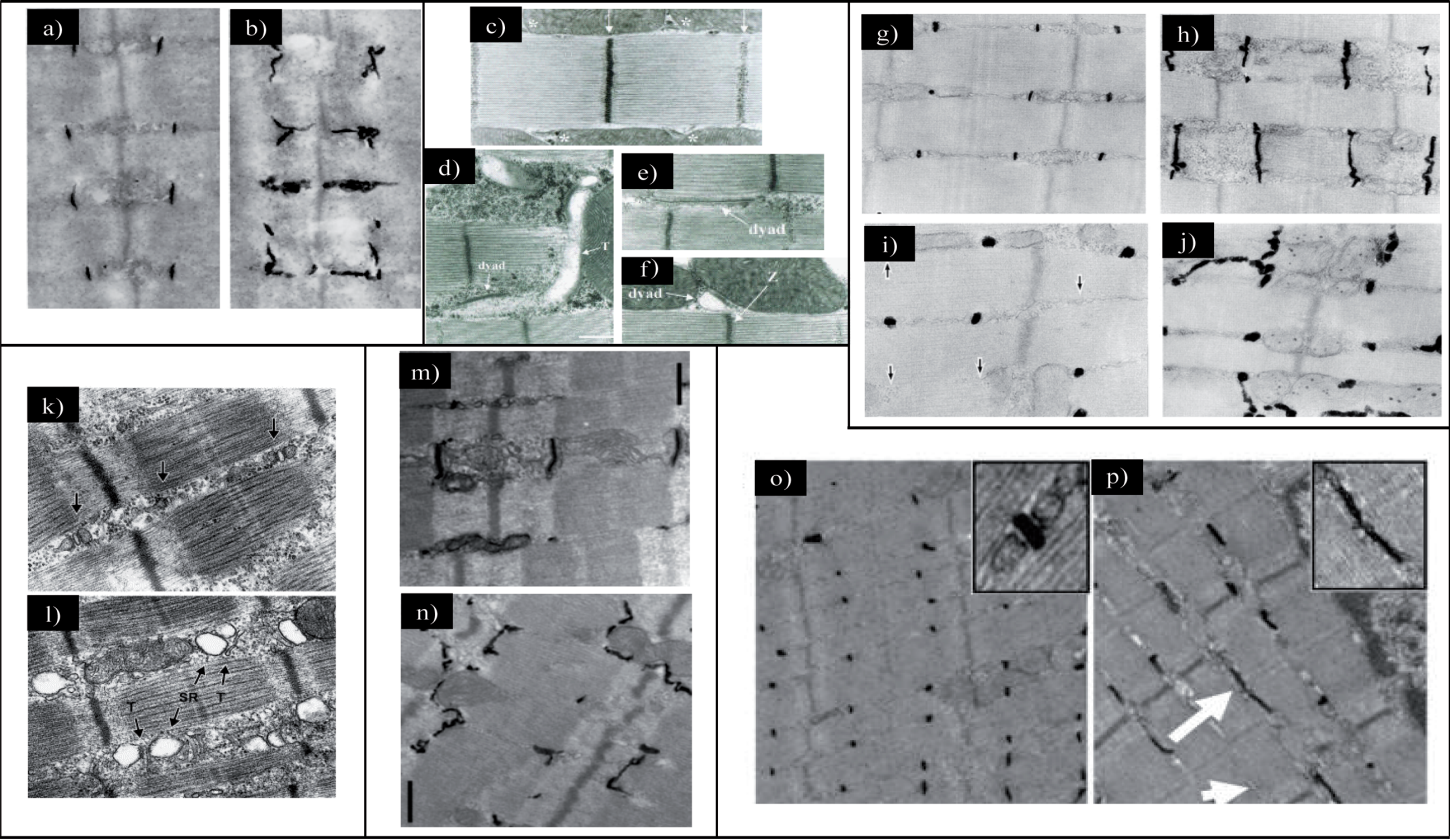
**Proteins implicated in triad organization in skeletal muscle**. **(a) **Electron microscopic image showing ferrocyanate-treated muscle from wild-type mouse. **(b) **Electron microscopic image showing ferrocyanate-treated muscle from *Cav3 *(caveolin 3)-mutant mouse. Note the altered appearance and orientation of T-tubules (from [32]; ^© ^2001 The American Society for Biochemistry and Molecular Biology). **(c) **Electron microscopic image from *Drosophila *normal muscle. Note that dyads are localized in proximity to the Z-line (similar to vertebrate). **(d through f) **Electron microscopic images from *Bin1 *(amphiphysin)-null *Drosophila *showing alteration of T-tubules resulting in **(d) **mislocalized, **(e) **longitudinal and **(f) **dilated tubules (from [43]; ^© ^2001 Cold Spring Harbor Laboatory Press). **(g through j) **Electron microscopic images of ferrocyanate-treated muscles from **(g and h) **wild-type and **(i and j) ***MG29 *(mitsugumin)-knockout mice demonstrating the alterations in T-tubule organization. Note the absence of **(i) **T-tubule or **(j) **longitudinal orientation (from [61]; ^© ^1999 The Rockefeller University Press. The Journal of Cell Biology, 2002, 159:695-705). **(k and l) **Electron microscopic images from **(k) **wild-type and **(l) ***JPH1 *(junctophilin)-knockout skeletal muscles from neonate mice showing the altered triads with swollen SR (reprinted from [73]; ^© ^2002 with permission from Elsevier). **(m and n) **Electron microscopic images of ferrocyanate-treated muscles from **(m) **wild-type and **(n) ***Dysf *(dysferlin)-knockout mice showing an abnormal shape of the T-tubule system (from [51]; ^© ^2010 John Wiley and Sons). **(o and p) **Electron microscopic images of ferrocyanate-treated muscles from **(o) **wild-type and **(p) ***Mtm1 *(myotubularin)-knockout mice revealing a severe alteration in T-tubule organization. Note the absence (arrowhead) or the longitudinally oriented (arrow) T-tubules (from [82]; ^© ^2009 National Academy of Sciences, USA).

Mice lacking CAV3 display a mild myopathic phenotype similar to the human pathology [32]. Besides, the ectopic expression of CAV3 in mice leads to Duchenne-like muscular dystrophic phenotype [33]. According to its localization at T-tubules, CAV3 deletion leads to disorganization of T-tubule membranes which become dilated and lose their transverse orientation (Figure 3a and 3b) [32]. This provides evidence that CAV3 is crucial for muscle function and has a role in T-tubules biogenesis. Several evidences lead to the hypothesis that similar mechanisms control the formation of the T-tubule system and the caveolae. Indeed, the mature and the developing T-tubules are associated with CAV3 and contain approximately four times more cholesterol than the plasma membrane [34,35]. Moreover, treatment of epithelial cells with Amphotericin B, a cholesterol-binding drug, results in a loss of morphologically defined caveolae at the cell surface. Similarly to CAV1 in epithelial cells, DHPR α and CAV3 are dramatically redistributed after Amphotericin B treatment of C2C12 myotubes. Interestingly, other cholesterol-rich compartments such as the trans-Golgi network do not seem affected [34].

#### Amphiphysin 2 (BIN1)

Mutations in *BIN1 *are associated to the autosomal recessive form of centronuclear myopathy [36], a disease characterized by muscle weakness, myofiber atrophy, and abnormal positioning of nuclei within muscle fibers.

Amphiphysins are membrane bending and curvature sensing proteins able to tubulate lipid membranes via their BAR (Bin/Amphiphysin/Rvs) domain. The ubiquitous amphiphysin 2, encoded by the *BIN1 *gene, is highly expressed in skeletal muscle and is proposed to participate in T-tubule biogenesis. Its role in this process is provided by a polybasic amino-acid stretch encoded by exon 11, which is important for its recruitment to T-tubule membranes [37,38]. This polybasic sequence can bind specifically the phosphoinositides PtdIns(4,5)*P*_2 _and PtdIns4*P in vitro*. Interestingly, the levels of PtdIns(4,5)*P*_2 _and BIN1 increase simultaneously during C2C12 myotubes differentiation [37,39].

In addition, BIN1 can tubulate membranes separately or in cooperation with dynamin 2 (DNM2), another protein mutated in centronuclear myopathy [40,41]. The cooperation between BIN1 and DNM2 is mediated by the interaction of BIN1 SH3 domain with the proline rich domain of DNM2. However, this interaction may not occur prior to BIN1 association to membranes, as the polybasic sequence binds to the SH3 domain when it is not membrane bound [42]. Indeed, PtdIns(4,5)*P*_2 _binding is necessary to release the SH3 domain, further enabling the interaction between the SH3 domain and DNM2 [42]. While the existence of this intramolecular regulation has been deciphered in cultured cells, it is not determined whether a similar mechanism regulates T-tubules curvature. Nevertheless, myotubes expressing a BIN1 mutant lacking the polybasic sequence failed to form a normal membrane tubules network [42].

A drosophila mutant lacking the orthologue of *BIN1 *(amphiphysin) exhibits a skeletal muscle defect associated to alterations in T-tubule morphology and EC coupling (Figure 3c-f) [43]. Interestingly, EM analysis of muscle biopsies from patients with *BIN1 *mutations revealed abnormal morphology of T-tubules [44]. In cultured COS-1 cells, overexpression of a mutation related to CNM in the BAR domain failed to form membrane tubules compared to the wild-type construct, suggesting that the lack of BIN1-mediated membrane remodeling could be at the basis of the muscle disease [36].

Surprisingly, no skeletal muscle defect was reported in *Bin1 *^-/- ^mice. However, these mice are dying perinatally due to cardiomyopathy, precluding a detailed analysis of skeletal muscle after birth [45].

#### Dysferlin

Dysferlin (DYSF) is a 230 kDa protein belonging to a family of genes similar to Caenorhabditis elegans ferlin. It contains a C-terminal transmembrane domain and multiple C2 domains implicated in calcium binding and calcium-dependent membrane fusion and repair. Mutations within the *DYSF *gene are associated with allelic muscular disorders including autosomal recessive limb-girdle muscular dystrophy type 2B [46], Miyoshi myopathy [47], and distal anterior compartment myopathy [48]. DYSF has a sarcolemmal localization in differentiated skeletal muscle, which is related to its role in membrane repair [49]. However, studies performed in C2C12 cells have shown that during myotubes differentiation, DYSF is associated to the T-tubules network in addition to sites of cells fusion, and can translocate to the sarcolemma upon myofiber injury [50]. Interestingly, studies performed in adult rat muscles, in which regeneration was induced by subcutaneous injection of notexin, have revealed that during early stage of muscle fiber regeneration (within the first week after notexin treatment), DYSF is mainly localized to T-tubules and translocates toward the sarcolemma in later stages of regeneration [51].

Several mouse lines have been generated to manipulate the level and the function of DYSF, and the spontaneous SJL strain was shown to encompass an in-frame deletion in the C-terminal of the *Dysf *gene [49,52-55]. Similarly to CAV3 mouse mutants, mice deficient for DYSF display alterations in T-tubule structure, with more dilated and longitudinally oriented tubules [51] (Figure 3m-n). These defects are considered as primary, as they are observed at early stage of the disease when abnormalities in the myofibrillar architecture and the sarcolemma are minimal. The role of DYSF in T-tubule biogenesis is still not determined, however, it has been suggested that DYSF contributes to the fusion of caveolin 3 containing vesicles with T-tubules. This suggestion is based on i) the interaction of DYSF with DHPR in mature skeletal muscle, ii) the known interaction between DYSF and CAV3, and iii) the partial co-localization between CAV3 and DYSF during early myogenesis [56,57]. This hypothesis is also supported by the accumulation of subsarcolemmal vacuoles contiguous with the T-tubule system in dysferlinopathy patients [58].

#### Mitsugumins

In addition to the proteins mentioned above, which are involved in the biogenesis of T-tubules, other proteins are implicated in the maturation of SR terminal cisternae, and the junction between T-tubules and SR. This is the case of members of the Synaptophysin family such as mitsugumin 29 (MG29 or synaptophysin-like 2, SYPL2), a transmembrane proteins highly enriched in heavy SR vesicles preparation [59]. *MG29 *is expressed early during myogenesis, even before the apparition of triads. It first associates to newly formed SR vesicles and then to triads, which appear later during myogenesis [59]. These observations implicate MG29 in the early formation of junctional SR and its connection to T-tubules [59,60]. In *MG29*-KO mice, decreased muscle mass and a slight decrease in the force generation capacity were observed [61]. Dysfunction of store-operated calcium entry (SOCE) leading to defects in intracellular calcium homeostasis, and increased muscle fatigability were also reported in these mice [62,63]. EM analysis of mutant muscles revealed morphological alterations in triad structures, including a swollen SR and longitudinal T-tubules (Figure 3g-j) [61]. However, the actual association between SR and T-tubules does not appear altered. It thus remains unclear whether such disorders in the triad structure have a link with the observed defect in SOCE.

Another mitsugumin, MG53 (also called TRIM72), has been identified as a key player in intracellular membrane trafficking and membrane repair machinery in striated muscles [64,65]. In addition to it specific expression in striated muscles, MG53 was shown to bind to dysferlin and caveolin 3, two proteins directly implicated in T-tubule biogenesis [64,66]. There are no studies demonstrating a direct implication of MG53 in the biogenesis of triad membranes; however, the current evidences sustain its implication as a potential new partner in this mechanism.

#### Junctophilins

JPH family members are identified as components of junctional membranes, where they may bridge the SR via their C-terminal transmembrane domain, with the T-tubule/plasma membrane via their N-terminal domain. More specifically, the N-terminal domain can bind to membrane phospholipids including sphingomyelin and phosphatidylcholine [67]. Among the four junctophilin-like proteins in mammals (JPH1-4), JPH1 is expressed mainly in skeletal muscle while JPH2 is also expressed in cardiac muscle and implicated in hypertrophic cardiomyopathies [67-69]. However, JPH3 and 4 are coexpressed in brain where JPH3 is found to be associated to a Huntington-like disease [70,71].

*JPH1 *deficient mice die shortly after birth due to defects in jaw muscles resulting in lack of milk suckling [72]. Electron microscopy analysis of skeletal muscle from embryos and newborn mice revealed several abnormalities in triad morphology, leading to defect in muscle contraction [72,73]. These anomalies include a reduced number of triads, swollen junctional SR, partially vacuolated longitudinal SR and irregular orientation of SR network (Figure 3k-l). Moreover, functional analysis of mutant muscles from neonate mice revealed that these muscles have an increased response to extracellular calcium stimuli, indicating a defect in EC coupling [72]. A specific role of JPH1 in the biogenesis of membrane junctions is also supported by its ability to induce junctions between the endoplasmic reticulum and the plasma membrane when overexpressed in amphibian embryonic cells [67]. In addition, the expression of *JPH1 *is enhanced after birth as an indicator of a role in the late biogenesis and maturation of triads [72].

#### Myotubularin

Myotubularin (MTM1) is part of a family of phosphoinositides phosphatases conserved through evolution down to yeast, and with 14 members in human [74]. While myotubularin is ubiquitously expressed, mutations within *MTM1 *lead to a skeletal muscle disorder: the X-linked form of centronuclear myopathy, also called myotubular myopathy, associating severe muscle atrophy and weakness at birth with abnormal positioning of nuclei [75]. This indicates that MTM1 has a muscle-specific role, which cannot be compensated by homologous proteins. *In vitro *and overexpression studies performed in yeast and mammalian cells have attributed to MTM1 a function in endosomal and membrane trafficking pathways and have shown that MTM1 specifically dephosphorylates PtdIns3*P *and PtdInsp(3,5)*P*_2 _into phosphatidylinositol and PtdIns5*P *respectively [76-79]. Recently, immunohistology and EM analysis revealed that AAV mediated overexpression of MTM1 in mouse muscle results in the formation of abnormal membrane structures [80]. These structures include vacuoles that are derived from sarcolemma and/or T-tubules, as they have positive staining for caveolin-3, dystrophin and dihydropyridine receptor (DHPR), and negative staining for laminin 2, and also contain the exogenous MTM1 protein [80]. This indicates a direct or indirect role for MTM1 in the generation or maintenance of membrane structures in skeletal muscle.

*Mtm1 *KO mice exhibit a progressive centronuclear myopathy [81]. EM analysis of *Mtm1 *KO muscles revealed alterations in T-tubules structure characterized by longitudinal orientation of T-tubules and the presence of triads deprived from T-tubule components (Figure 3o-p) [82]. These alterations become more pronounced with disease progression [44,82]. Similarly, *mtm1 *zebrafish morphants and *mtm*-depleted drosophila muscles display structural defects of the triads, and such defects have been also observed in muscles from patients with myotubular myopathy [44,83,84]. Since MTM1 expression is increased in the postnatal life in mouse and *Mtm1 *KO muscles display less altered T-tubules at an early stage than at a late stage of the disease, it is likely that MTM1 plays a key role in the late maturation and/or the maintenance of T-tubules rather than in their early biogenesis.

#### The RyR-DHPR complex

The RyR-DHPR interaction is physically linking the SR to T-tubules in skeletal muscle, and thus mediates the translation of the action potential into intracellular calcium release. RyR1, the skeletal muscle ryanodine receptor, is implicated in the susceptibility to malignant hyperthermia [85,86], and mutated in myopathies with variable histological outcomes as central core [87,88], multi-minicore [89], congenital fiber type disproportion [90,91], and/or nuclei centralization [92,93]. It was thought that the direct interaction between RyR and DHPR is necessary for T-tubule and SR assembly [94]. The concomitant expression of RyR and DHPR during myogenesis is consistent with this idea [94]. However, it has been shown by immunofluorescence and EM studies performed in mouse models lacking one or both proteins that it is unlikely to be the case. More specifically, mutant mice lacking RyR (dyspedic) or DHPR (muscular dysgenesis, mdg mouse), or even both proteins form triadic junctions with a similar architecture than wild-type mice [95-99]. Moreover, these myotubes have a normal disposition of other SR components such as calsequestrin and triadin, although calcium release from intracellular stores is greatly affected [96,100]. This indicates that neither RyR nor DHPR are necessary for the biogenesis of the triad structure.

In addition, the targeting of DHPR and RyR to their respective membrane is independent of each other [18,99]. This suggests that the T-tubule and SR separately posses the potential for self-assembly.

## Conclusion

**Figure 4:**
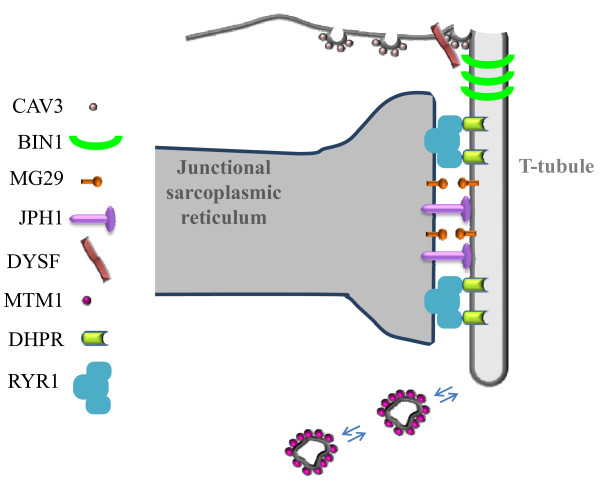
**Hypothetical model for triad biogenesis in skeletal muscle**. The different proteins discussed in the section Molecular mechanisms involved in T-tubule biogenesis and triad formation and maintenance are indicated, and their hypothetical implications in T-tubule biogenesis and triad formation and maintenance are depicted. Details can be found in the conclusion section.

Whether T-tubule origin is from an inward or outward movement of membranes has been debated for many decades. Some co-workers have suggested that caveolar invagination fuses with the internal membrane tubules and thus facilitates their connection to the surface [34]. However, increasing evidences support the hypothesis that T-tubules are formed by surface membrane invaginations starting from caveolae [17,18,37]. During myofiber maturation, the T-tubule network grows up to occupy the entire muscle fiber. It is likely that T-tubule proliferation happens by two complementary mechanisms: 1) membrane invagination and tubulation, and 2) membrane addition (Figure 4). BIN1 is a promising candidate for the regulation of membrane tubulation at T-tubules. Invagination of T-tubules may be triggered at the site of caveolae formation on the sarcolemma. It is also possible that the growth of T-tubules is ensured by new membranes derived from endosomes and/or caveolae. CAV3, DYSF and MTM1 may play a major role in the formation and remodeling of growing T-tubules by regulating the incorporation of internal membranes and/or the turnover of existing tubular membranes. On the SR side, mitsugumin and junctophilin proteins would be important for the positioning of junctional SR to the proximity of transversal tubules. Membrane invagination and tubulation might be more active during early stages of T-tubule biogenesis (i.e. during muscle differentiation or after muscle injury), and membrane addition may be the main mechanism for T-tubule proliferation during postnatal muscle growth or maintenance.

It is likely that other proteins may have important roles in these processes, like triadin [19,101,102], a protein located on the SR, or telethonin (TCAP)) a large protein implicated in sarcomere assembly and recently suggested to play a role in T-tubule biogenesis [103], although their direct impact in triad biogenesis in skeletal muscle remains to be confirmed. There is still a lot to learn about other potential proteins involved in triad biogenesis and to understand their biological role in muscle development.

## Competing interests

The authors declare that they have no competing interests.

## Authors' contributions

LAQ and JL conceived and drafted the manuscript.
